# OASIS +: leveraging machine learning to improve the prognostic accuracy of OASIS severity score for predicting in-hospital mortality

**DOI:** 10.1186/s12911-021-01517-7

**Published:** 2021-05-13

**Authors:** Yasser EL-Manzalawy, Mostafa Abbas, Ian Hoaglund, Alvaro Ulloa Cerna, Thomas B. Morland, Christopher M. Haggerty, Eric S. Hall, Brandon K. Fornwalt

**Affiliations:** 1 Department of Translational Data Science and Informatics, Geisinger, Danville, PA 17822 USA; 2 College of Information Sciences and Technology, Pennsylvania State University, University Park, PA 16802 USA; 3 Department of General Internal Medicine, Geisinger, Danville, PA 17822 USA; 4 Department of Radiology, Geisinger, Danville, PA 17822 USA

**Keywords:** In-hospital mortality prediction, Point-based severity scores, Critical care outcomes, Supervised machine learning

## Abstract

**Background:**

Severity scores assess the acuity of critical illness by penalizing for the deviation of physiologic measurements from normal and aggregating these penalties (also called “weights” or “subscores”) into a final score (or probability) for quantifying the severity of critical illness (or the likelihood of in-hospital mortality). Although these simple additive models are human readable and interpretable, their predictive performance needs to be further improved.

**Methods:**

We present OASIS +, a variant of the Oxford Acute Severity of Illness Score (OASIS) in which an ensemble of 200 decision trees is used to predict in-hospital mortality based on the 10 same clinical variables in OASIS.

**Results:**

Using a test set of 9566 admissions extracted from the MIMIC-III database, we show that OASIS + outperforms nine previously developed severity scoring methods (including OASIS) in predicting in-hospital mortality. Furthermore, our results show that the supervised learning algorithms considered in our experiments demonstrated higher predictive performance when trained using the observed clinical variables as opposed to OASIS subscores.

**Conclusions:**

Our results suggest that there is room for improving the prognostic accuracy of the OASIS severity scores by replacing the simple linear additive scoring function with more sophisticated non-linear machine learning models such as RF and XGB.

**Supplementary Information:**

The online version contains supplementary material available at 10.1186/s12911-021-01517-7.

## Background

In the past three decades, several severity scores have been developed with the primary objective of predicting in-hospital mortality from clinical and/or biological measurements, often collected within the first 24 h of admission to the intensive care unit (ICU) admission [1, 2]. Point-based severity scores compute the severity of an illness by modeling the deviations of a set of clinical and/or biological variables from their normal physiologic values [2]. More specifically, point-based severity scores rely on linear or non-linear transformations of the measurements into subscores from which a final score is computed as a linear sum. Based on this approach, several severity scoring methods have been proposed including acute physiology and chronic health evaluation (APACHE) [3–6], Simplified Acute Physiology Score (SAPS) [7–10], Logistic Organ Dysfunction Score (LODS) [11], systemic inflammatory response syndrome (SIRS) [12], Sequential Organ Failure Assessment (SOFA) [13], and Oxford Acute Severity of Illness Score (OASIS) [14]. These severity scoring methods have been widely used in many research and clinical applications including predicting mortality, length of stay (LoS), stratifying patients for clinical trials, and evaluation of ICU quality of care [15].

Early prediction of mortality in ICU patients can improve health outcomes and is essential for timely interventions by ICU clinicians [16]. Regardless of the availability of many severity scoring methods, the prognostic performance of these models in predicting in-hospital mortality remains far from satisfactory [17, 18]. Since these severity scoring methods can be viewed as logistic regression models, one promising direction for boosting their predictive performance is to replace the simple additive model with more sophisticated supervised machine learning algorithms such as random forest (RF) [19] or eXtreme gradient boosting (XGB) [20]. Unfortunately, the expected improvement in performance might introduce a tradeoff in model interpretability resulting from the increased model complexity which might also hamper clinicians’ ability to associate explanations with the predictions made by the model.

Against this background, our preliminary objective is to accentuate the promise and demonstrate the feasibility of developing a new generation of severity scoring methods based on state-of-the-art non-linear supervised learning algorithms. Specifically, we present OASIS +, a novel method for predicting in-hospital mortality using an ensemble of non-linear decision trees trained using the same 10 clinical variables used to calculate the OASIS score. By restricting our model to these 10 variables, we provide a predictive model with a minimal set of variables that are frequently measured during the ICU stay and have low rates of missing values. Using a test set of ICU stays lasting at least for 24 h that represent 9566 distinct adult patients, we show that OASIS + outperforms nine previously developed severity scoring methods (including OASIS) in predicting in-hospital mortality. Our major contributions are as follows: (1) We introduce an improved variant of the OASIS severity score with substantial improvements in predictive and prognostic performance; (2) We assess the performance of nine severity scores on predicting in-hospital mortality and demonstrate superior performance of OASIS + over all of them; (3) We show that OASIS thresholds for transforming clinical measurements into subscores are not optimal for the MIMIC-III data; (4) We release the implementation of the OASIS + model as an online tool for predicting in-hospital mortality as well as the associated Python scripts for benchmarking OASIS + using independent test data from other health systems.

## Related work

Since its introduction in 2013 as a de-identified publicly available dataset for supporting the secondary analysis of critical care data, the MIMIC-III database [21] has been extensively used for developing machine learning based models for a variety of prediction tasks [22], including predicting in-hospital mortality. Such models can be categorized based on different criteria related to the type of the machine learning algorithm (shallow vs. deep learning algorithms), the type of the input variables (scalar vs. temporal), and the target patient population (all ICU patients vs. a sub-cohort of the ICU patients such as septic patients).

Shallow machine learning algorithms such as random forests (RF) [19], eXtreme gradient boosting (XGB) [20], and support vector machine (SVM) [23] have been used in developing in-hospital mortality prediction models (e.g., [24, 25]) or as baseline models for comparisons with the proposed deep learning models (e.g., [26, 27]). Several deep learning models for multivariate time series based on conventional neural networks (CNN) [28], long-short terms memory (LSTM) [26, 27], and gated recurrent unit (GRU) networks [26] have been proposed and shown to have superior performance over shallow models as well as simple severity scores.

Generally, the MIMIC-III database has three types of data: (1) static variables such as age, gender, and admission type; (2) temporal (sequence) data such as vital signs and laboratory measurements; (3) unstructured data in the form of clinical notes. Shallow models often aggregate time series data collected over the first 24 or 48 h of admission into scalar variables, while deep learning models for sequence classification accept time series data as inputs. Zhang et al. [27] proposed two hybrid deep learning architectures for learning from all three types of data.

Several in-hospital mortality prediction models have been evaluated using all adult ICU patients (e.g., [26, 27]), while others have been evaluated for a sub-population of the ICU patients. Lin et al. [25] proposed a RF model for predicting in-hospital mortality for ICU patients with acute kidney injury (AKI). Kong et al. [24] evaluated four shallow machine learning algorithms on predicting in-hospital mortality for sepsis patients in the ICU.

Recent efforts in developing machine learning algorithms for in-hospital mortality prediction for ICU patients have also focused on supporting the reproducibility and the interpretability of the developed models. Models reproducibility had been mainly supported via sharing the source code with the scientific community in public repositories. Interpretability of the models have been supported via techniques for reporting or visualizing feature importance [28]. Despite these efforts, simple severity scores (e.g. APACHE and SOFA) remain commonly used in real-world settings for supporting real-time decision making and for characterizing Electronic Health Records (EHR) datasets used for research. For example, during the COVID-19 pandemic, enormous research papers have used the SOFA score for characterizing the datasets (e.g., [29–31]). In addition, some researchers assessed the predictive performance of the SOFA score for predicting in-hospital mortality for COVID-19 patients [32, 33]. Moreover, novel severity scores for COVID-19 patients have been developed [34–36]. We believe that two important factors can in part justify the lack of using deep learning models in the ICU settings (and healthcare settings in general): (1) lack of trust in ‘black-box’ models [37, 38], especially deep learning models published without associated techniques for interpretating how the model work or for explaining the model predictions; (2) implementing a severity score is far easier than deploying a deep learning model based on its source code and scripts for retrieving and pre-processing of the raw input test data. To support the usability of machine learning predictive models in healthcare settings, we argue the developers of these models to share their learned models as online calculators or as standalone software components (e.g., Docker containers [39]) and, therefore, enable easy deployment of their models to be used and validated by other researchers.

## Methods

### Ethics statement

The retrospective cohort training and test datasets were extracted from the Medical Information Mart for Intensive Care III (MIMIC-III) database (version 1.4) [21]. MIMIC-III is a publicly available database of 46,476 ICU patients hospitalized in Beth Israel Deaconess Medical Center (BIDMC) and the public access to the de-identified database has been approved by the BIDMC and MIT institutional review boards [21]. All data processing and analyses presented in this study have been conducted in accordance with MIMIC-III guidelines and regulations.

### Study population

MIMIC-III contains clinical data for 49,785 hospital admissions associated with 38,597 adult patients admitted to ICU between 2001 and 2012. The following criteria were used to exclude patients from our cohort dataset: (1) age less than 18 years or greater than 90 years; (2) ICU stays with duration less than 24 h. For patients with multiple ICU stays, we included the first ICU stay with LoS greater than or equal to 24 h. The primary outcome for our analysis was in-hospital mortality. Our final dataset included 31,884 distinct patients and ICU stays. We randomly partitioned the data into 70% and 30% for training and testing, respectively. Missing variables were imputed as normal [40] such that their corresponding missing subscores were ZEROs.

### Data extraction

We downloaded MIMIC-III files from https://mimic.physionet.org/. Then, we followed the instructions from the MIMIC code repository [41] to build a local PostgreSQL [42] database and adapted MIMIC-III code to extract our dataset and compute patient comorbidities and first day severity scores. For supporting reproducibility of our work, the PostgreSQL query script is provided in the supplementary material (see Additional file 2).

### Point-based severity scores

Since their presentation in the 1980s, point-based severity scores (e.g., APACHE-II, SAPS-III, OASIS) have been commonly used in the ICU settings for assessing disease severity in critically ill patients and predicting poor health outcomes such as in-hospital or 30-day mortality [15]. Many severity scores are calculated using clinical variables (e.g., temperature and heart rate) and biological variables (e.g., white blood cell count) collected from the first day in the ICU (see Additional file 1: Table S1). Other severity scores such as (SOFA) [13] can be computed repetitively every day or every time new measurements are presented.

A common characteristic among point-based severity scores is that they are based on logistic regression models and, therefore, can be viewed as multivariate additive linear models for predicting the severity of critical illness. Such models can be easily computed and interpreted by a physician. Briefly, each variable is transformed into a subscore such that a subscore of ZERO indicates that the measured variable is within its normal range and higher subscores penalize for observed variables outside their normal range (or value). These subscores are determined using a consensus opinion or data-driven approaches [1]. An overall severity score is then computed as the sum of all subscores. The higher the overall score, the greater the disease burden and the higher likelihood of poor health outcomes. For example, let's consider the OASIS severity score [14]. This score is computed using 10 clinical variables from first day in the ICU. Using Table 1, each clinical variable is transformed into a corresponding subscore and the OASIS score is the sum of these 10 subscores. An OASIS probability of mortality is also computed using $\frac{1}{1+{e}^{-(0.1275x-6.1746)}}$ where $x$ is the OASIS score.

**Table 1:** Mapping OASIS clinical variables into subscores

Variable	Range	Subscore	Variable	Range	Subscore
Age (years)	< 24	0	Temperature (°C)	< 33.22	3
	24–53	3		33.22–35.93	4
	54–77	6		35.94–36.39	2
	78–89	9		36.40–36.88	0
	> 90	7		36.89–39.88	2
GCS	3–7	10		> 39.88	6
	8–13	4	Urine output (cc/day)	< 671	10
	14	3		671–1426.99	5
	15	0		1427–2543.99	1
Heart rate (per minute)	< 33	4		2544–6896	0
	33–88	0		> 6896	8
	89–106	1	PreICU LoS (hours)	< 0.17	5
	107–125	3		0.17–4.94	3
	> 125	6		4.95–24.00	0
Mean blood pressure (mmHg)	< 20.65	4		24.01–311.80	2
	20.65–50.99	3		> 311.80	1
	51–61.32	2	Ventilated?	No	0
	61.33–143.44	0		Yes	9
	> 143.44	3	Elective surgery?	No	6
Resp. rate (per minute)	< 6	10		Yes	0
	6–12	1			
	13–22	0			
	23–30	1			
	31–44	6			
	> 44	9			

In the present study, we assessed the predictive performance of nine previously developed severity scores (summarized in Additional file 1: Table S1) for predicting in-hospital mortality using data collected during the first ICU day.

### Machine learning models

We experimented with three widely used supervised machine learning algorithms: (1) Random Forest [19] with 200 trees (RF200); (2) eXtreme gradient boosting [20] with 200 weak tree learners (XGB200); (3) Logistic Regression (LR) [43] with L2 regularization. These algorithms are implemented in the Scikit-learn machine learning library [44]. We accepted all the default settings for the hyperparameters except for the number of decision trees which we set it to 200. The LR model is a linear and human interpretable model while RF200 and XGB200 models are ensembles of 200 non-linear decision trees. To get insights into how these ensemble models work, we used feature importance scores to quantify the contribution of each feature to the predictions made by both RF200 and XGB200.

### Statistical analysis and performance evaluation

Categorical variables are reported as percentages and the chi-square test was used to assess whether two (or more) proportions are different from each other. Continuous variables are summarized as medians and interquartile ranges (IQR) and the non-parametric Mann–Whitney test was used to determine the differences in the distribution of a variable (e.g., Age) in survival and non-survival groups. All statistical analyses were performed using R version 3.6.2 [45] and a *p* value less than 0.05 was considered significant.

The predictive performance of the machine learning models was assessed using five commonly used predictive performance metrics [46]: Accuracy (ACC), Sensitivity (Sn); Specificity (Sp); Matthews correlation coefficient (MCC); and Area Under the receiver operator Curve (AUC) [47]. The prognostic performance of different models is assessed using calibration curves [48] and the root-mean-square error (RMSE) is used to quantify calibration errors [49].

## Results

### Characteristics of train and test sets

Basic summary statistics of the training and test sets are provided in Table 2 and Additional file 1: Table S2, respectively. Both tables show that the following variables are significantly associated with in-hospital mortality in ICU patients: increased age, increased length of stay, increased number of pre-existing conditions (i.e., comorbidities), and increased severity of the critical illness (using any of the nine pre-existing severity scores considered in our analysis). Although this exploratory analysis suggests that any randomly selected patient from the non-survival group is likely to have severity scores higher than those for a randomly selected patient from the survival group, it is of particular interest to assess how well these severity scores can discriminate between patients within each of the two groups.

**Table 2:** Summary statistics of MIMIC-III training data

Variable	Survivals (*n* = 19,953)	Non-survivals (*n* = 2365)	*p* value
Age (years)	64.09 (51.39–75.54)	71.32 (58.56–80.46)	< 0.001
LoS (days)	2.36 (1.55–4.31)	4.38 (2.17–8.93)	< 0.001
No. of comorbidities	3 (2–5)	4 (2–5)	< 0.001
*Ethnicity*			
African	1504 (7.54%)	121 (5.12%)	< 0.001
Asian	484 (2.43%)	61 (2.58%)	
Hispanic	667 (3.34%)	42 (1.78%)	
White	14,217 (71.25%)	1596 (67.48%)	
Others	3081 (15.44%)	545 (23.04%)	
*Gender*			
Female	8320 (41.7%)	1065 (45.03%)	< 0.01
Male	11,633 (58.3%)	1300 (54.97%)	
APS-III	37 (28–48)	59 (43–77)	< 0.001
LODS	3 (2–5)	6 (4–8)	< 0.001
MLODS	2 (1–4)	4 (2–6)	< 0.001
OASIS	30 (24–36)	39 (33–45)	< 0.001
QSOFA	2 (1–2)	2 (2–2)	< 0.001
SAPS	17 (14–21)	22 (18–25)	< 0.001
SAPS-II	32 (24–40)	47 (37–58)	< 0.001
SIRS	3 (2–4)	3 (3–4)	< 0.001
SOFA	3 (2–5)	6 (4–9)	< 0.001

Table 3 characterizes the 10 clinical variables used for computing OASIS severity scores in the training and test sets. For both datasets, all clinical variables (except pre-ICU LoS) were found to be drawn from significantly different distributions for survival and non-survival groups. It should be noted that the non-parametric Mann–Whitney test is a rank sum test that ranks all of the observations from each group and then sums the ranks from one of the groups which is compared with the expected rank sum. Therefore, it is possible for the two groups to have significantly different rank sums while their medians are equal as we noted for the Glasgow Coma Score (GCS) and Pre-ICU LoS variables.

**Table 3:** Summary statistics of OASIS variables in train and test sets

Variable	Train (*n* = 22,318)			Test (*n* = 9566)		
	Survivals (*n* = 19,953)	Non-survivals (*n* = 2365)	*p* value	Survivals (*n* = 8560)	Non-survivals (*n* = 1006)	*p* value
Age	64 (51–75)	71 (58–80)	< 0.001	63 (51–75)	71.5 (60–80)	< 0.001
GCS	15 (14–15)	15 (13–15)	< 0.001	15 (14–15)	15 (12–15)	< 0.001
Heart rate	101 (90–115)	111 (95–128)	< 0.001	101 (90–115)	110 (94–126)	< 0.001
Mean blood pressure	59.67 (52.67–86)	55 (47–82)	< 0.001	59 (53–86)	55 (47–81.21)	< 0.001
Respiratory rate	26 (18.5–30)	28 (24–33)	< 0.001	26 (18–30)	28 (24–33)	< 0.001
Temperature	36.17 (35.7–37.5)	36 (35.44–37.5)	< 0.001	36.17 (35.72–37.5)	36.06 (35.5–37.71)	< 0.001
Urine output	1895 (1245–2770)	1189 (645–2050)	< 0.001	1900 (1235–2785)	1167 (640.5–1972.5)	< 0.001
Pre-ICU-LoS	0 (0–18)	0 (0–23)	< 0.001	0 (0–17)	0 (0–27)	0.12
ventilated?: Yes	9723 (48.73%)	1570 (66.38%)	< 0.001	4186 (48.9%)	676 (67.2%)	< 0.001
Elective surgery?: Yes	3357 (16.82%)	76 (3.21%)	< 0.001	1367 (15.97%)	38 (3.78%)	< 0.001

Additional file 1: Table S3 summarizes the baseline characteristics of Elixhauser comorbidities [50] for the survival and non-survival groups in the training and test datasets. Out of the 30 Elixhauser comorbidities, 18 comorbidities in the training set and 16 comorbidities in the test set have significantly different proportions of survivals and non-survivals. The top three most frequent comorbidities are: Hypertension (more frequent in survivals), Fluid and Electrolyte Disorders (more frequent in non-survivals), and Cardiac Arrhythmias (more frequent in non-survivals).

### Assessment of nine severity scores on predicting in-hospital mortality

**Fig. 1:**
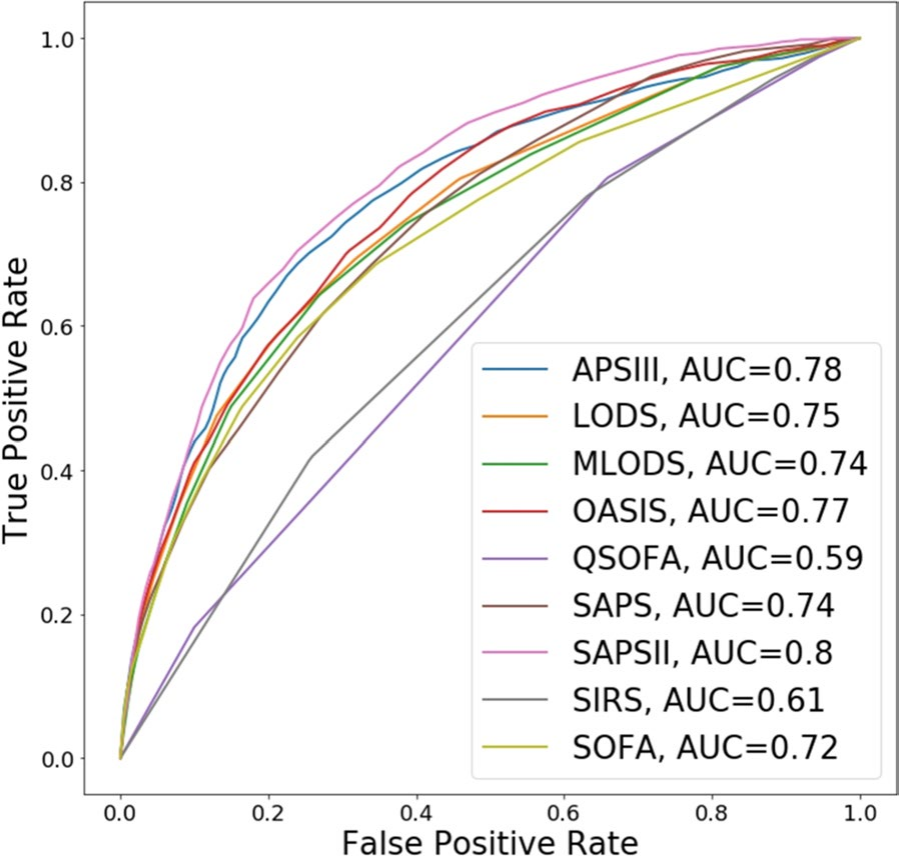
Performance (in terms of ROC curves and associated AUC scores) of nine severity scores estimated using MIMIC-III test set for predicted in-hospital mortality

We evaluated the performance of nine previously developed severity scores (Additional file 1: Table S1) on predicting in-hospital mortality using the MIMIC-III test set. Table 4 presents the performance of these nine severity scores using five widely used metrics. Since every scoring method has different scale, we normalized each score in the range [0, 1] and we used the MIMIC-III training set to estimate the optimal threshold for transforming the scores into binary labels (i.e., survivals vs. non-survivals). The optimal threshold was computed by maximizing the Youden's J statistic [51]. Figure 1 shows the Receiver Operating Curve (ROC) curves and corresponding AUC scores for each of the nine severity score methods. We found that models with the best performance (i.e., $AUC \in [0.77{-}0.8]$) were based on OASIS, APS-III, and SAPS-II. SOFA, MLODS, SAPS, and LODS demonstrated moderate performance (i.e., $AUC \in [0.72{-}0.75])$, and SIRS and QSOFA performed poorly with AUC scores of 0.61 and 0.59, respectively. Not only did the SAPS-II model have the highest AUC, but its ROC curve demonstrated superior performance (in terms of threshold-dependent metrics) at all possible thresholds compared to the ROC curves for the other eight severity models.

**Table 4:** Performance comparisons of nine severity score models for predicting in-hospital mortality estimated using MIMIC-III test set

Method	ACC (%)	Sn	Sp	MCC	AUC	Threshold
APSIII	89.4	0.16	0.98	0.24	0.78	0.27
LODS	89.0	0.17	0.97	0.23	0.75	0.30
MLODS	89.3	0.12	0.98	0.19	0.74	0.24
OASIS	69.4	0.70	0.69	0.25	0.77	0.49
QSOFA	39.1	0.81	0.34	0.10	0.59	0.67
SAPS	71.6	0.62	0.73	0.23	0.74	0.48
SAPSII	88.0	0.29	0.95	0.28	0.80	0.35
SIRS	20.4	0.94	0.12	0.06	0.61	1.00
SOFA	88.6	0.17	0.97	0.21	0.72	0.27

Of the top three severity scores, SAPS-II and APS-III scores are computed using 14 and 20 variables, respectively (See Additional file 1: Table S1), and OASIS score is computed using 10 variables (See Table 1). Therefore, among these three scores, OASIS score: uses the smallest number of variables; uses no biological variables; and has the lowest performance on predicting in-hospital mortality. In what follows, we present a novel variant of OASIS score, OASIS +, that leverages non-linear machine learning supervised algorithms for outperforming the SAPS-II and APS-III models on predicting in-hospital mortality.

### OASIS + outperforms all nine severity scores on predicting in-hospital mortality

**Fig. 2:**
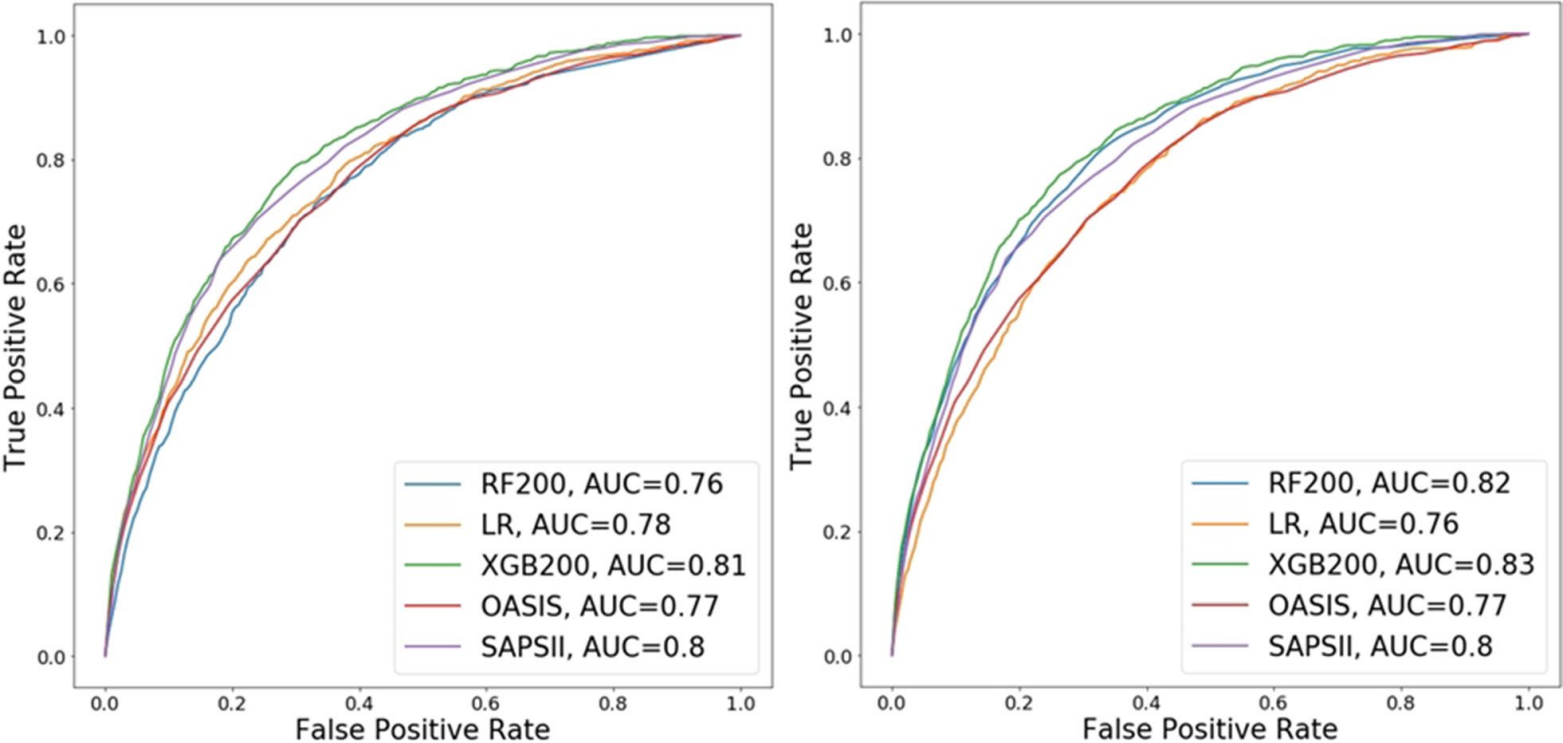
Performance (in terms of ROC curves and associated AUC scores) of three machine learning models for predicting in-hospital mortality trained using oasis score and subscores (left) and oasis variables (right)

We considered two approaches for developing supervised learning classifiers using the 10 clinical variables used for computing OASIS scores (Table 1). In the first approach, we used the 10 subscores and OASIS probability as input features. In the second approach, we used the 10 clinical variables (without transforming them into subscores) as input features. In both cases, we evaluated one linear supervised learning algorithm, LR, and two algorithms for building ensembles of 200 non-linear decision trees, RF200 and XGB200. The predictive performance of these six models is summarized in Fig. 2 and Table 5. Using OASIS subscores, the best performing model, XGB200, has an AUC score of 0.81 while models based on LR and RF200 slightly outperform OASIS score. Using OASIS variables, RF200 and XGB200 have AUC scores of 0.82 and 0.83, respectively. The results show that the two ensemble learning algorithms achieve better performance when trained using the measured values of the clinical variables as opposed to their non-linear transformation derived from OASIS benchmark dataset [14]. This observation suggests that OASIS non-linear transformations in Table 1 are more likely to be data-specific and might not generalize well to other patient populations. The main interesting observation is that a 0.06 improvement in AUC score is obtained by replacing the OASIS linear additive scoring function with the non-linear XGB model trained using non-transformed clinical variables (hereafter called OASIS + model).

**Table 5:** Performance comparisons of different machine learning models for predicting in-hospital mortality estimated using MIMIC-III test set

Features	Method	ACC (%)	Sn	Sp	MCC	AUC	Threshold
OASIS subscores	RF200	77.5	0.54	0.80	0.25	0.76	0.16
	LR	73.9	0.66	0.75	0.28	0.78	0.12
	XGB200	70.9	0.79	0.70	0.31	0.81	0.10
OASIS variables	RF200	88.0	0.34	0.94	0.31	0.82	0.33
	LR	70.0	0.69	0.70	0.25	0.77	0.10
	XGB200	72.8	0.78	0.72	0.33	0.83	0.10

**Fig. 3:**
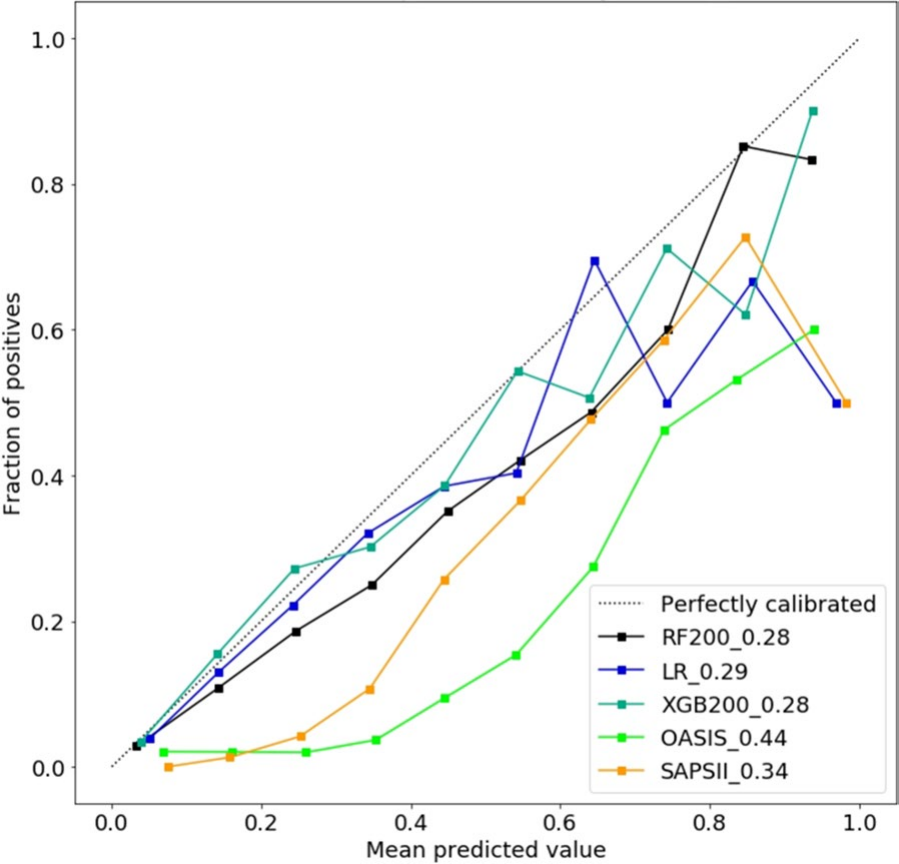
Calibration curves assessing the consistency between the actual risk and predicted risk of different models

Since OASIS + is a prognostic model for predicting the risk of in-hospital mortality, it should be evaluated in terms of discrimination (e.g., using AUC and threshold-dependent metrics) as well as calibration [49, 52]. Figure 3 shows the calibration curves for SAPS-II, OASIS, and the three machine learning models trained using OASIS variables. We found that OASIS and SAPS-II have higher estimated calibration errors when compared with our three machine learning models. The lowest estimated calibration error is observed for XGB200 (OASIS +) and RF200 models.

**Fig. 4:**
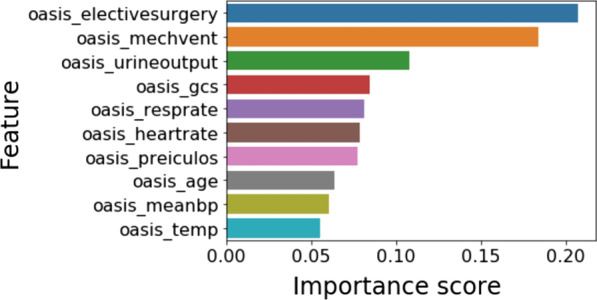
Features importance scores of the OASIS + model

Figure 4 shows the contribution of each variable in the predictions made by OASIS + model also called OASIS + feature importance scores. Comparing OASIS + feature importance scores (Fig. 4) with OASIS non-linear transformations (Table 1) reveals several discrepancies between relative variable importance of the two models. For example, in OASIS + , the three variables with the highest feature importance scores are: elective surgery, ventilation use, and urine output. On the other hand, the three OASIS variables with the highest weights (i.e., OASIS subscore of 10) are: GCS in the range 3–7, respiratory rate less than 6 per minute, and urine output less than 671 cc/day. Unlike OASIS +, each variable in OASIS can take different weights with a ZERO weight corresponding to normal measurements. Thus, for a given case, respiratory rate might have the highest contribution (subscore of 10) to the computed OASIS score while in another case respiratory rate might be in the normal range (13–32) and have a ZERO contribution to the OASIS score.

**Fig. 5:**
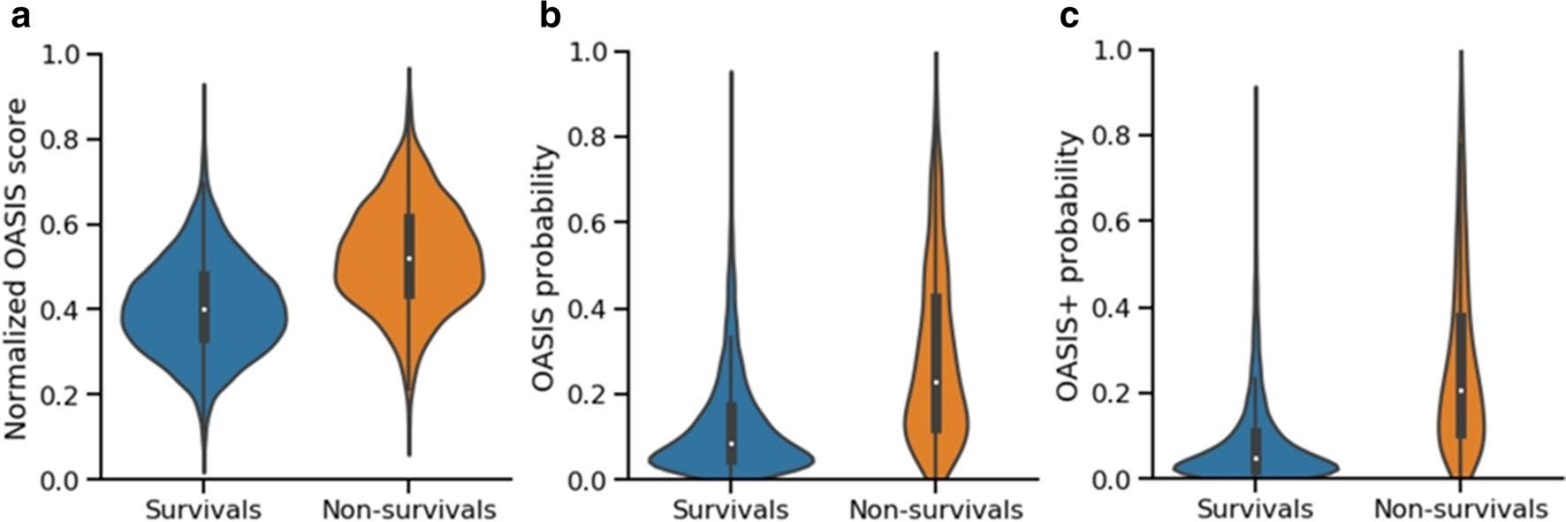
Violin plots, for **a** normalized OASIS scores, **b** OASIS probabilities, **c** OASIS + probabilities in survivals and non-survivals groups, computed using the MIMIC-III test set

Figure 5 shows the violin plots, estimated using the test set, for normalized OASIS scores, OASIS probabilities, and OASIS + predicted probabilities in survivals and non-survivals groups. A violin plot shows both a box plot and a rotated kernel density plot. We noted that OASIS scores follow a normal distribution. This acknowledges the same finding reported in [14] on a different patient population. In the three cases, the median score or probability in the non-survival group is higher than the corresponding median in the survival group. However, the largest and most significant (*p* value equals 2.4e−250) difference between the two medians is observed for OASIS + probabilities.

**Fig. 6:**
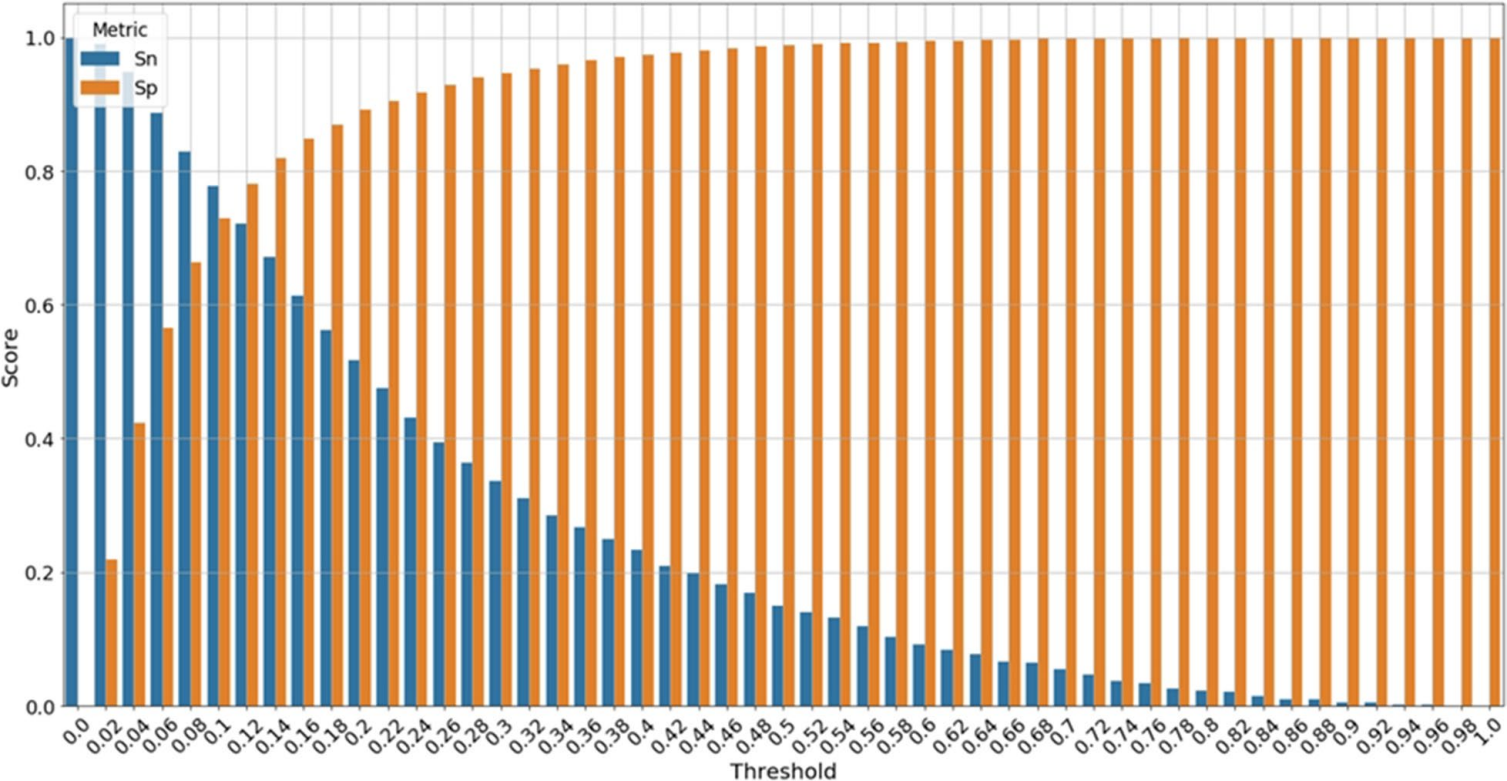
Trade-off between sensitivity and specificity for different choices of the threshold for discretizing the continuous predicted probability into a predicted binary label

Finally, Table 5 compares the performance of the six predictive models, considered in this experiment, using AUC and four threshold-dependent metrics. For all the models, we noticed that the optimal threshold, estimated using the training data only, for transforming the model predicted probability into a binary label is lower than 0.5. This is an artifact of the uneven ratio of non-survivals to survivals since the number of survival cases is almost 10 times the number of non-survivals. Figure 6 shows the tradeoff between sensitivity and specificity in OASIS + for different choices of the probability threshold. At a threshold equals 0.1, the sensitivity and specificity of the model are 0.78 and 0.72, respectively. The highest MCC of 0.36 is reached at a threshold of 0.13 which yields 0.70 and 0.80 sensitivity and specificity, respectively. The complete set of OASIS + results at different thresholds is provided in Additional file 1: Table S4.

### Performance improvements using more features

**Fig. 7:**
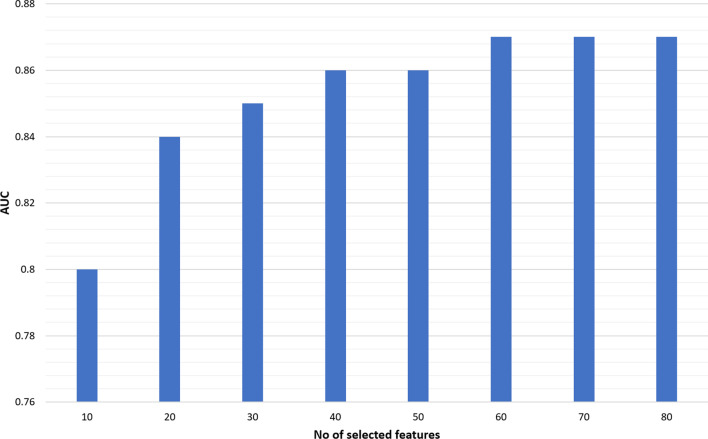
Test performance (in terms of AUC scores) of the XGB200 classifiers trained using $k$ selected features

To facilitate the utility of OASIS + as an online severity score calculator, our design goal is to minimize the number of variables that the user has to manually input to get the predictions. However, it is of interest to identify how much improvement can be obtained using additional features. To address this question, we considered 84 variables representing all the subscores used in the nine severity scores. We then evaluated the three machine learning algorithms trained using $k=\{\mathrm{10,20},\dots ,80\}$ features. For feature selection, we used RF feature importance (RFFI) scores [19] estimated from the training data. We found that XGB200 consistently outperformed the other classifiers. Figure 7 shows the AUC scores estimated using the test data when the XGB200 model was trained using the top $k$ selected features. The highest observed AUC score was 0.87 using at least 60 variables.

### OASIS + web application

We deployed the OASIS + model using the streamlit framework, https://github.com/streamlit/streamlit, and made it publicly available at https://oasis-score.herokuapp.com/. The web app provides an interface for the user to input the measured values for the 10 clinical variables and select the values of the threshold for transforming OASIS + predicted probabilities of in-hospital mortality into a binary label (e.g., deceased vs. survived). The displayed results include: OASIS severity score and probability; interpretation of OASIS score; OASIS + predicted probability and predicted in-hospital mortality; OASIS + model interpretation in terms of feature importance scores quantifying the contribution of the 10 clinical variable to the prediction made by the XGB200 classifier (see Fig. 4); and a projection of the user-specified threshold on the ROC curve of the OASIS + indicating sensitivity and 1-specificity of OASIS + model at the user-specified threshold estimated using MIMIC-III test set.

## Discussion

The present study introduced, OASIS +, a novel machine learning-based model for predicting in-hospital mortality using the 10 clinical variables used in the OASIS severity score [14]. To the best of our knowledge, this is the first work to consider using OASIS variables and/or subscores for developing severity score prediction models. Our results suggest that there is room for improving the prognostic accuracy of traditional severity scores by replacing the simple linear additive scoring function with more sophisticated non-linear machine learning models such as RF and XGB. Our results also suggest that the two non-linear supervised learning algorithms considered in our experiments can be directly trained using the observed clinical variables without the need for transforming these measurements into subscores.

Over the past two decades several severity scoring systems were subjected to continuous refinements and improvements. For example, SAPS and APACHE scores have three and four versions, respectively. A common pattern in these severity scoring methods with multiple versions is that newer versions often have more variables than those used in preceding versions. For example, SAPS scores versions I–III are based on 14, 17, and 20 variables, respectively. Another example is APACHE scores versions I–IV which use 38, 12, 20, and 145 variables, respectively. While adding more relevant physiologic measurements is likely to improve the predictive performance of a severity scoring methods, it makes implementation for use in real-time challenging. Ideally, refinement and improved performance of existing scoring systems could be achieved without adding more variables. This objective can be reached using more sophisticated machine learning algorithms. To the best of our knowledge, OASIS + is the first study that demonstrates the promise of focusing on the machine learning component of a severity scoring system to significantly improve its predictive performance. This opens up the possibility for improving existing severity scoring methods by adapting the approach presented in this work.

OASIS + shares several of the advantages of OASIS when compared to other severity scores. Both OASIS and OASIS + are based on fewer variables than the vast majority of severity scores. Also, these variables are frequently measured during the ICU stay and do not have high rates of missing values. Another major advantage of the OASIS score is that it can be computed manually without the need for informatics support. However, because it uses an ensemble of complex 200 decision trees, OASIS + cannot be computed manually. To facilitate easy internet-based access to an OASIS + calculator for individual ICU patients, we have deployed an OASIS + model as a freely accessible web app for scientific use. For batch computations on large-scale data, we shared OASIS + model and supplementary Python scripts on a public source code repository which can be accessed at https://bitbucket.org/i2rlab/oasis/src/master/. Therefore, future studies can easily benchmark the performance of OASIS + using datasets from other health systems.

Taken the availability of large-scale datasets in healthcare systems together with the recent advances in machine learning research, we argue that population-specific severity prediction models should be preferred over traditional severity scoring methods. We believe this for the following reasons. First, our results showed that MIMIC-III specific machine learning models using only 10 clinical variables outperformed nine commonly used severity scoring methods. Several related studies (e.g., [24, 25, 53]) have also shown that machine learning models outperform severity scores on predicting in-hospital mortality. Second, developing health system specific (or local) prediction models enables continuous improvements of the model by including more training data (as more data become available), adding new clinical or laboratory variables to the model, or re-training the model using newly developed machine learning algorithms.

Several machine learning based predictive models are treated as “black-boxes”, which are systems that hide their internal logic to the user [54]. Such models often have better predictive accuracy compared with interpretable models such as linear additive models [55]. However, relying on sophisticated non-linear machine learning models trained using large-scale biomedical datasets raises concerns regarding the ethics and trustability of the utility of black-box decision making systems in ICU settings and healthcare in general [56, 57]. These concerns could be addressed in part by using techniques for post-hoc interpretation and explanation of the predictive model [54]. To shed some light on how the OASIS + model works, we used the XGB200 inferred feature importance scores to quantify the contribution of each of the 10 clinical measurements to the predictions made by OASIS +. We have also inspected the model via the application of sensitivity analysis to examine the effect of the OASIS + threshold parameter on the sensitivity and specificity of the model. Ultimately, we believe that machine learning predictive models should be held to the same standard as medications or other therapies given to patients. If using a predictive model improves patient outcomes during a prospective clinical trial (for example where patients are randomized to either having the model results available or not), then the model should be adopted clinically regardless of whether the model is fully transparent or “black-box”. As evidence of this, a class of drugs known as beta blockers have been proven to prolong life when given to certain categories of patients with heart failure, but the underlying physiology of these drugs and exactly how they improve survival remain poorly understood [58–60]. Despite this lack of understanding of their mechanism of action, beta blockers are widely used due to their proven efficacy. We believe that machine learning models should be held to the same standard.

The present study has some limitations. First, this is a retrospective study that used a dataset from a single health system. Second, the data used for training our models were extracted from the same source as the test dataset. Third, the inclusion of severity scores in our experiments was restricted by the availability of PostgreSQL scripts for computing these scores from MIMIC-III in the MIMIC code repository [41]. Our ongoing work aims at evaluating the generalizability of OASIS + on an independent validation set and including other commonly used severity scores such as APACHE-IV [6] in the analysis.

## Conclusions

We have presented a novel machine learning based severity prediction model, OASIS +. OASIS + is a variant of the OASIS severity score, where the non-linear transformation of the input clinical variables is omitted, and the simple additive function is substituted with an ensemble of 200 non-linear decision trees. Thus, using a machine learning approach we were able to enhance OASIS + model performance without the need for introducing additional variables beyond the 10 readily available variables used in the OASIS model. In addition to the original OASIS score, OASIS + outperformed eight other severity scores in predicting in-hospital mortality. The improved OASIS + performance came with a trade-off in human readability and cannot be computed manually. To address these limitations, we used feature importance and sensitivity analysis to improve the interpretability of the model and we deployed the model as a publicly available web server to enable access to the model. Moreover, we supported the application of OASIS + model to large-scale datasets by sharing the learned model and necessary Python scripts through a source code repository. Our future work aims at: improving the explanation of OASIS + model and its predictions; and evaluating OASIS + using an independent test set.

## Supplementary Information

**Additional file 1.** Supplementary Tables S1–S4.

**Additional file 2.** PostGreSQL script for retrieving data from the MIMIC-III database

## Data Availability

The datasets presented in the current study are available in the MIMIC III database (https://physionet.org/content/mimiciii/1.4/). Our machine learning model and associated Python scripts are freely available at https://bitbucket.org/i2rlab/oasis/.

## Abbreviations

ACC: Accuracy
AKI: Acute kidney injury
APACHE: Acute physiology and chronic health evaluation
AUC: Area under the receiver operator curve
BIDMC: Beth Israel Deaconess Medical Center
CNN: Conventional neural networks
EHR: Electronic health records
XGB: EXtreme gradient boosting
GRU: Gated recurrent unit
GCS: Glasgow Coma Score
ICU: Intensive care unit
IQR: Interquartile ranges
LoS: Length of stay
LODS: Logistic Organ Dysfunction Score
LR: Logistic regression
LSTM: Long-short terms memory
MCC: Matthews correlation coefficient
MIMIC-III: Medical Information Mart for Intensive Care III
OASIS: Oxford Acute Severity of Illness Score
RF: Random forest
RMSE: Root mean square error
Sn: Sensitivity
SOFA: Sequential organ failure assessment
SAPS: Simplified Acute Physiology Score
Sp: Specificity
SVM: Support vector machine
SIRS: Systemic inflammatory response syndrome
